# Estimation of Ambient Dose Equivalent Rate Distribution Map Using Walking Survey Technique in Hirosaki City, Aomori, Japan

**DOI:** 10.3390/ijerph20032657

**Published:** 2023-02-01

**Authors:** Worawat Poltabtim, Saowarak Musikawan, Arkarapol Thumwong, Yasutaka Omori, Chutima Kranrod, Masahiro Hosoda, Kiadtisak Saenboonruang, Shinji Tokonami

**Affiliations:** 1 Department of Radiation Science, Graduate School of Health Sciences, Hirosaki University, 66-1 Honcho, Hirosaki 036-8564, Aomori, Japan; 2 Institute of Radiation Emergency Medicine, Hirosaki University, 66-1 Honcho, Hirosaki 036-8564, Aomori, Japan; 3 Special Research Unit of Radiation Technology for Advanced Materials (RTAM), Faculty of Science, Kasetsart University, Bangkok 10900, Thailand; 4 Department of Materials Science, Faculty of Science, Kasetsart University, Bangkok 10900, Thailand; 5 The Research Center for Safety, Metrology, and Nuclear Quality Technology (PRTKMMN), Research Organization for Nuclear Energy, National Research and Innovation Agency of Indonesia (BRIN), JI. Lebak Bulus Raya No. 49, Jakarta Selatan 12440, DKI Jakarta, Indonesia; 6 Department of Applied Radiation and Isotopes, Faculty of Science, Kasetsart University, Bangkok 10900, Thailand

**Keywords:** gamma radiation, ambient dose equivalent rate, distribution map, walking survey technique

## Abstract

At present, much emphasis is placed on the health risks associated with radioactivity present in the environment, especially since the accident at the Fukushima Daiichi Nuclear Power Plant. In this study, a walking survey was conducted in Hirosaki City using a NaI(Tl) scintillation spectrometer to estimate and map the distribution of the ambient dose equivalent rate to monitor the radiological safety of the general public in Hirosaki City, where many nuclear facilities are located nearby. The average (±standard deviation) ambient dose equivalent rate was 0.056 ± 0.020 µSv h^−1^. By comparison with the measurement data, it was found that the values of 85% of the data obtained using the walking survey technique deviated within ±20% relative to those obtained by spot measurements. Furthermore, the distribution of dose rates obtained in the nighttime survey was not significantly different from those obtained in the daytime.

## 1. Introduction

In March 2011, an accident at Fukushima Daiichi Nuclear Power Plant (FDNPP) released large quantities of artificial radionuclides that increased the ambient dose equivalent rates in the environment. Since the accident, the interest of ordinary citizens in environmental radiation has grown, and some are concerned about personal radiation exposure dose levels from the environment [1]. Hirosaki University is one of the universities that measured the radiation dose levels around the campus and Hirosaki City after the FDNPP accident [2,3] to monitor and estimate the radiological hazard to public health. Although only absorbed dose rates at low levels were observed at Hirosaki University after the FDNPP accident (comparable to the natural background radiation level) [3], radiation measurements should be regularly recorded to build data on the amount of chronic general public exposure to natural radiation, such as cosmic rays, radon, and terrestrial gamma rays on a daily basis, because exposure to natural background radiation is one of the most significant parts of their total exposure to radiation. The regular measurements are more important for expanding cities and developing countries because rapid urbanization alters the radiation environment. Usually, the building materials that have been used in the expansion elevate radiation levels [4,5,6].

**Figure 1:**
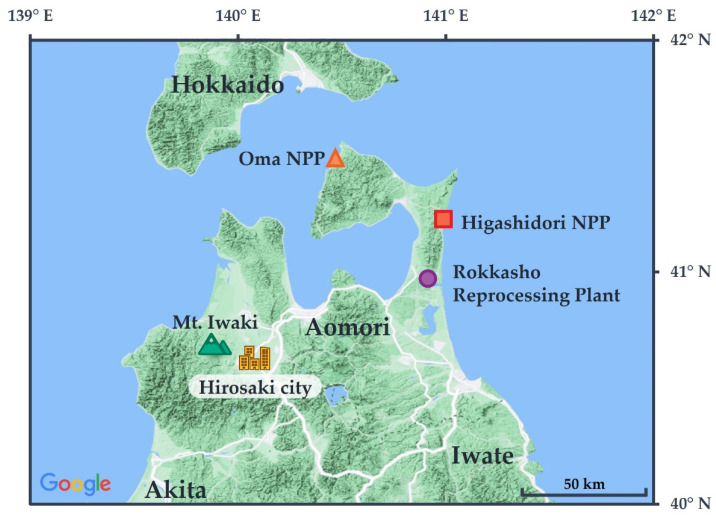
Map of Aomori Prefecture and locations of Hirosaki City, nuclear facilities, and Mt. Iwaki.

Measurements of the ambient dose equivalent rate that related to natural background radiation in Hirosaki City can be used to develop a radioactivity database to respond to and to communicate with the public who are concerned about radiation, because exposure to natural background radiation is one of the most significant parts of their total exposure to radiation. Moreover, there are several nuclear facilities located near Hirosaki City, as shown in Figure 1. Two nuclear power plants (NPPs) and a commercial nuclear fuel reprocessing plant are located in the northern part of Aomori Prefecture, about 100 km from Hirosaki City.

Ambient dose equivalent rates were measured in this study to monitor the baseline of natural radiation doses to the general public in Hirosaki City. A walking survey in Hirosaki City, mainly in the Hirosaki University and Castle Park areas, was carried out to develop a detailed distribution map of ambient dose equivalent rates. Additionally, a comparison was carried out using the obtained data between the walking survey technique and spot measurements. The accuracy of the walking survey was quantified to evaluate whether the walking survey technique is applicable. Furthermore, the effect of daytime and nighttime on the distribution of dose rates was investigated in this study.

## 2. Materials and Methods

### 2.1. Outline and Operation of Walking Survey Technique

**Figure 2:**
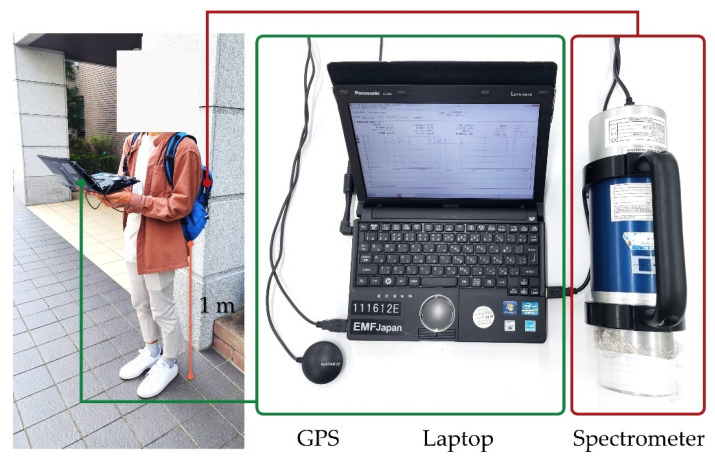
Walking survey system with the GPS and spectrometer carried in the backpack.

The walking survey was performed using a gamma-measuring system consisting of a 3 in × 3 in cylindrical sodium iodide (NaI(Tl)) scintillation spectrometer (EMF-211; EMF Japan Co. Ltd.; Himeji, Japan) connected to a portable laptop computer for central data processing and a global positioning system (GPS) receiver (G-STAR IV model BU-353S4; GlobalSat WorldCom Corp.; New Taipei City, Taiwan) to record location coordinates at each measuring point. A typical configuration for a walking survey setup is shown in Figure 2. The NaI(Tl) spectrometer has been widely used for the detection of ionizing radiation, especially gamma rays originating from nuclei of radioactive elements. The spectrometer was installed in a nylon backpack and carried on the operator’s back, approximately 1 m above the ground. During the walking survey, the gamma count rates and the GPS location data (latitude and longitude; precision 10 m) were recorded simultaneously at a fixed time interval of 12 s along the route, based on the precision of the GPS receiver at 10 m and a walking speed of the operator at around 5 km h^−1^. Previous research [7] examined the accuracy and spatial resolution of dose rate mapping at a measurement interval of 12 s and walking speed of 5 km h^−1^ (average walking speed for Japanese) in a relatively high dose rate area in Fukushima, and it showed good accuracy and spatial resolution.

Since the gamma counts were measured using a spectrometer inside the backpack and close to the operator’s body, readings were adjusted by multiplying a correction factor based on body-shielding of the spectrometer in the backpack. The body-shielding correction factor (*CF_body-sheilding_*) was estimated by making spot measurements inside and outside the backpack on the operator’s back at 10 measurement points in the main campus (Bunkyo campus) of Hirosaki University and applying an associated correction factor that equated to the count rates inside the backpack. Following correction, the ambient dose equivalent rates were calculated using a dose rate conversion factor (*DCF*) based on the radiation response of the spectrometer to the reference survey meter (1 in × 1 in cylindrical NaI(Tl) scintillation, TCS-171; Hitachi, Ltd.; Tokyo, Japan), based on spot measurements at 10 points on the Bunkyo campus. Notably, these portable NaI(Tl) scintillation survey meters have been generally used in projects of mapping the ambient dose equivalent rate distribution in Fukushima. This survey meter was calibrated using a ^137^Cs source. The calibration factor was 0.98 for a dose rate range below 1 µSv h^−1^. At the same time, the ambient dose equivalent rates were processed together with location information by using free software, Generic Mapping Tools (GMT) [8], and mapped using the Google Earth software.

### 2.2. In Situ Gamma Pulse-Height Distribution

At the spot measurement points, in situ gamma-ray pulse-height distributions were obtained using a 3 in × 3 in NaI(Tl) scintillation spectrometer (EMF-211). Measurements were conducted 1 m above the ground at each measurement point on 14 September 2022. The counting time was set to 900 s. A response matrix (22 × 22) unfolded the pulse-height distribution of gamma rays into the energy spectrum of gamma-ray flux, and then dose contributions for each radionuclide were calculated according to previous reports to discriminate between natural and artificial radionuclides [9,10,11].

### 2.3. Survey Areas

The walking surveys were performed in typical public outdoor areas of Hirosaki City (streets, yards, and gardens), mainly on the Hirosaki University Bunkyo campus and in Hirosaki Castle Park. On the Bunkyo campus, spot measurements were also performed using the reference survey meter (TCS-171) at 52 measurement points around the campus. For the spot measurements using the survey meter, the ambient dose equivalent rate was measured at 1 m above the ground in four different directions at a single point, with the time constant of a measurement being 30 s. The spot locations were recorded as coordinates using the Google Maps application on a smartphone. The surveys were conducted during 14–15 September 2022. The weather was sunny, with clear sky throughout the entire daytime and nighttime measurement periods. The temperature, humidity, and pressure during the surveys were monitored using a Thermo Recorder, TR-73U (T&D Corporation; Tokyo, Japan). The average (±standard deviation) results for temperature, humidity, and pressure were 26.6 ± 1.4 °C, 57.8 ± 4.0%, and 1009.9 ± 1.5 hPa, respectively.

## 3. Results and Discussions

### 3.1. Body-Shielding Correction Factor

**Figure 3:**
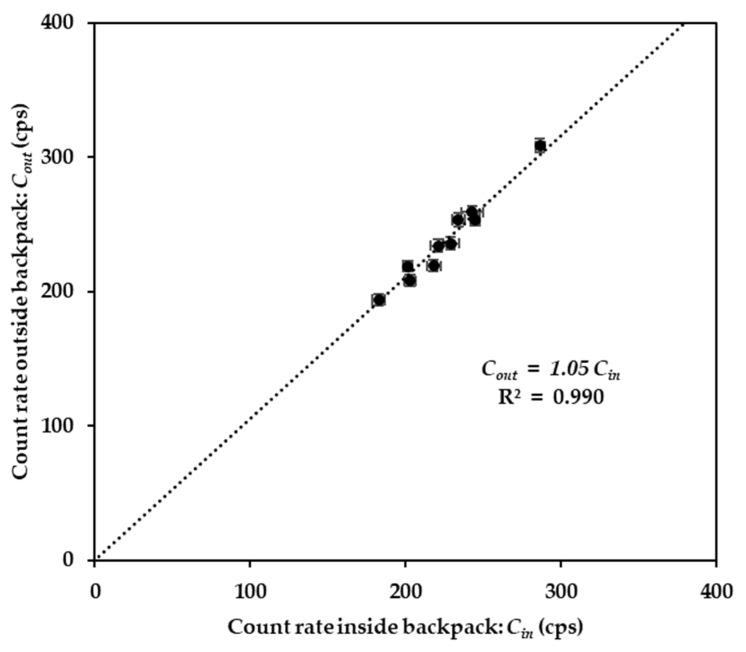
Correlation between count rates outside and inside the backpack.

Since the count rates from the spectrometer were measured inside the backpack, it was necessary to estimate the shielding factor of the backpack and the operator’s body toward gamma rays in order to represent the unshielded external dose rates. The correlation between the count rates inside the backpack (carried on the operator’s back) and outside (placed on a tripod) is shown in Figure 3. The *CF_body-shielding_* and the standard uncertainty values were 1.05 and 0.01, respectively. Thus, the count rate in air (*C_out_*) at each measurement point was estimated using Equation (1):

(1) $$C_{out}={1.05C}_{in}$$

Using this estimated value, the median and mean values of calculated *C_out_* were about 5.4% larger than the equivalent measured *C_in_.* Similar results were reported by Ramzaev et al. [12], who used a similar NaI(Tl) spectrometer, with the placement of the spectrometer in a backpack on a human reduced the total ambient dose equivalent rate by about 12% for background measurements at typical outdoor locations, and about 3–4% reduction due to scattered photons from ^137^Cs contamination in the environment. The operator- and backpack-related reduction effects can be explained by two interrelated processes that occur with the gamma rays passing through the human body and backpack. First is attenuation of primary photons, and the second is buildup of photons with low energy. The latter process substantially compensates a reduction in detection rate for scattered radiation from the environment [12]. As the result, the mean values of count rate inside the backpack are 5% lower than those from outside the backpack. The difference between the two measurements is statistically significant. Therefore, the reduction effects should be considered and used during the walking survey to expect the lower impact from body-shielding.

**Figure 4:**
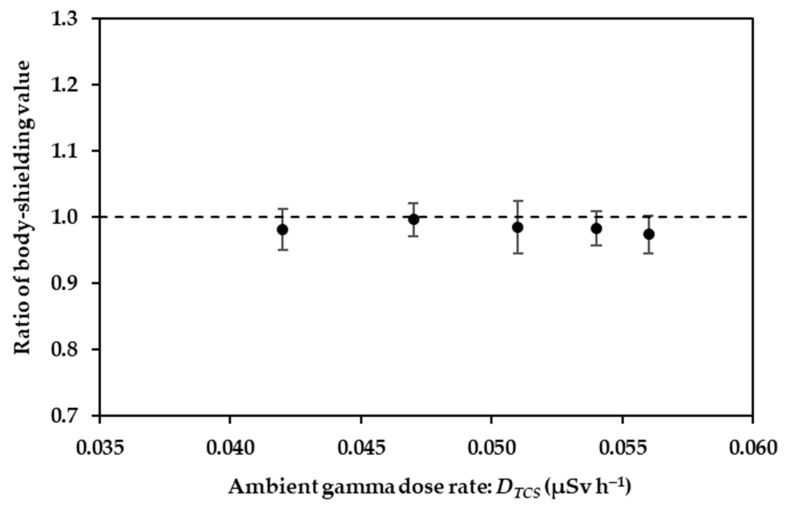
Scatter plot showing the ratio of two-operator body-shielding values and ambient dose equivalent rate.

The body-shielding values of two operators were compared. Figure 4 shows the scatter plots between the ratio of the body-shielding values for two operators and the ambient equivalent dose rates using the survey meter TCS-171 (*D_TCS_*). The ratios approached 1 (0.98 ± 0.03). Therefore, no significant difference was found between different operators who performed the walking survey in the current study; hence, no correction was required. However, some research indicated that the body-shielding values depended on the physical parameters of the operator, such as body weight and height [13].

### 3.2. Ambient Dose Equivalent Rate Conversion Factor

**Figure 5:**
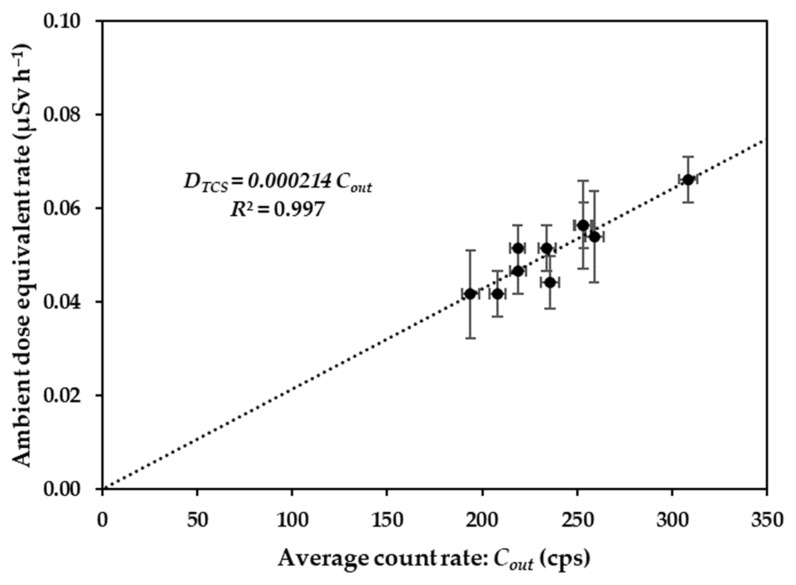
Relationship between the EMF spectrometer count rates calibrated with ambient dose equivalent rates obtained using the survey meter.

In general, the EMF spectrometer provides the data results in terms of the count rate. The dose conversion factor (*DCF*) is required to convert the count rate to the ambient dose equivalent rate. The *DCF* was examined at 10 points on the Bunkyo campus by comparing the results obtained from the spectrometer with those from the reference survey meter. Figure 5 shows the scatter plot of the ambient dose equivalent rate and the average count rate obtained from the spot survey. The slope of this regression line was used as the *DCF* and was 2.14 × 10^−4^ (µSv h^−1^ cps^−1^) with a standard uncertainty of 4.01 × 10^−6^. Based on the *DCF* and *CF*_body-shielding_, the ambient dose equivalent rate (*D_out_*: µSv h^−1^) at 1 m above the ground surface was calculated using Equation (2):

(2) $$D_{out}={1.05C}_{in}\times 2.14\times 10^{-4}$$

The standard uncertainty of the calculated ambient dose equivalent rate can be determined as a combined relative standard of the measured value. The standard uncertainty of the measured value from the EMF spectrometer was 10% at 20 µSv h^−1^ or less [14]. Relative standard uncertainties for the body-shielding correction factor and the ambient dose equivalent rate conversion factor were 0.8% and 1.9%, respectively. Therefore, the estimation value of the combined relative expanded uncertainty (*k* = 2) for the calculated ambient dose equivalent rate from Equation (2) was 20.4%.

### 3.3. Distribution of Ambient Dose Equivalent Rate

**Figure 6:**
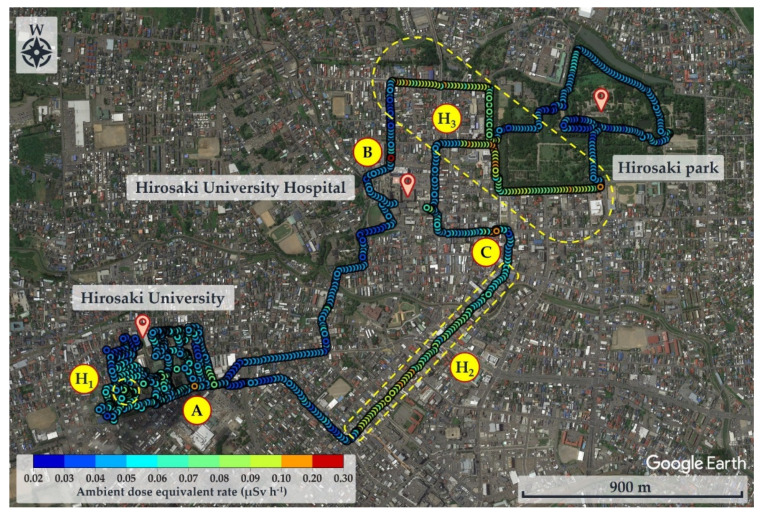
Distribution map of ambient dose equivalent rate in air for Hirosaki City.

The distribution map of the ambient dose equivalent rate along the walking survey route in Hirosaki City is shown in Figure 6. This route was drawn based on 1055 data using the GMT software. A heterogeneous distribution of ambient dose equivalent rates was observed. The ambient dose equivalent rates in this study were in the range 0.022–0.213 µSv h^−1^ with an average value of 0.056 ± 0.020 µSv h^−1^. The distribution map of the absorbed dose rate in air obtained from the walking survey in Hirosaki City was established and is shown in Appendix A. The absorbed dose rates in air were in the range 0.007–0.146 µGy h^−1^, with an average value of 0.032 ± 0.015 µGy h^−1^. 

**Figure 7:**
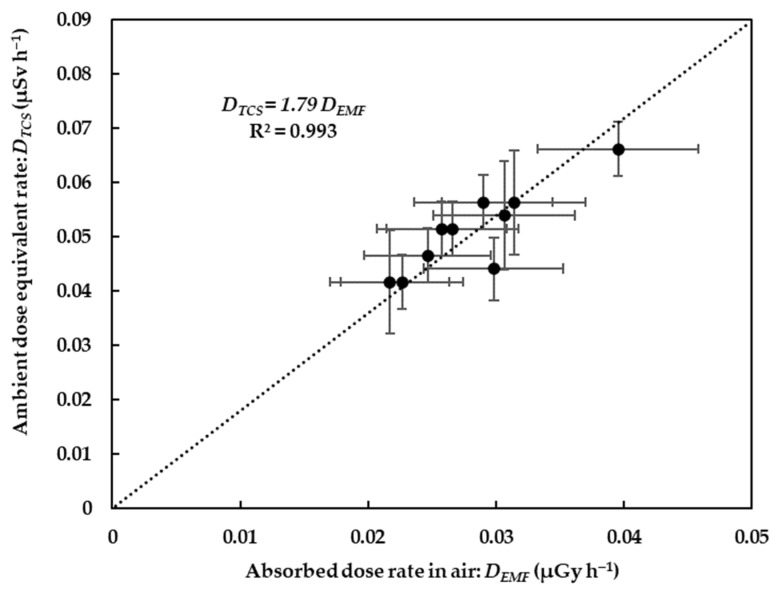
Scatter plot of correlation between absorbed dose rate in air calculated using software and response matrix method using the spectrometer (EMF-211) and ambient dose equivalent rate obtained using the survey meter (TCS-171).

Figure 7 shows comparisons of the absorbed dose rates in air with the ambient dose equivalent rates obtained from the measurements carried out in Hirosaki City. The results clearly showed that the absorbed dose rates in air correlated well with the ambient dose equivalent rates, with the slope of the regression line being about 1.79 ± 0.05 Sv Gy^−1^. A similar value (1.63 Sv Gy^−1^, with a standard error: 0.02 Sv Gy^−1^) was reported by Omori et al. [15] for outdoor sites in Fukushima City, Japan. Ramzaev and Barkovsky [16] reported an average ratio of 1.47 Sv Gy^−1^ for wooden houses and asphalt streets. 

According to UNSCEAR (2000) [17], the world’s absorbed dose rates in air are in the range 0.024–0.160 µGy h^−1^, with an average of 0.057 µGy h^−1^, which is higher than the average value obtained in the current study. The average value in the current study was also lower than the average absorbed dose rate reported from a Japanese nationwide survey, for which the average, maximum, and minimum absorbed dose rates in air were estimated to be 0.050, 0.147, and 0.022 µGy h^−1^, respectively [18]. 

The radiation doses from natural background radiation, particularly from terrestrial radiation in Japan are relatively low compared to other major world cities, especially in Aomori Prefecture, because the geology of Aomori Prefecture consists of loam and other material deposits containing low-level natural radionuclides that are widely distributed throughout the prefecture, including Hirosaki City [19]. Low average dose rates were reported in other studies. For example, Iyogi et al. [20] reported an average dose rate of 0.028 µGy h^−1^ for Aomori Prefecture. In 2013, Yoshino et al. [2] carried out a gamma-ray dose rate survey in Hirosaki City using pocket survey meters. They reported an average dose rate of 0.031 ± 0.008 µGy h^−1^ along the main streets, while in Hirosaki Park it was 0.023 ± 0.003 µGy h^−1^. An average dose rate of 0.024 ± 0.003 µGy h^−1^ was observed by Hosoda et al. [3] from a spot survey on the Bunkyo campus of Hirosaki University. 

Figure 6 shows relatively high ambient dose equivalent rates of over 0.060 µSv h^–1^, compared to natural background radiation in Japan distributed in the H_1_, H_2_, and H_3_ areas. In the H_1_ area, ambient dose equivalent rates were in the range 0.060–0.079 µSv h^−1^ with an average value of 0.069 ± 0.006 µSv h^−1^, observed near buildings that were decorated with granite. Similarly, high ambient dose equivalent rates in the H_2_ (average value of 0.079 ± 0.016 µSv h^−1^) and H_3_ (average value of 0.088 ± 0.012 µSv h^−1^) areas were observed on sidewalks covered with granite slabs. Furthermore, some hotspot locations (very high dose rate locations ≥ 0.12 µSv h^−1^) were observed during the current walking survey (Figure 6 and Table 1), such as the maximum dose rate of 0.213 µSv h^−1^ at location B (near the nameplate of the Medical School; GPS: 140.464467° E, 40.599320° N), while a dose rate of 0.144 µSv h^−1^ was recorded at location A (near the University nameplate beside the main entrance gate of Hirosaki University; GPS: 140.473865° E, 40.589883° N), and a dose rate of 0.125 was observed at location C (GPS: 140.469362° E, 40.602588° N). At or near these hotspot locations there are structures or decoration with granite, as shown in Table 1. To ensure that the high dose rates were the result of the granite material, a Geiger–Müller (GM) survey meter (TGS-1146; Hitachi, Ltd.; Tokyo, Japan) was used to estimate the net count rate from β-emitters at distances of 0.5 cm and 100 cm from the granite material around these hotspot locations. Additionally, surface activity was also calculated using the net count rate obtained at 0.5 cm from the wall surface. The surface activity can be calculated based on the net count rate divided by the equipment efficiency (0.475), source efficiency (assumed to be 0.5), and effective area (19.6 cm^2^). As shown in Table 1, the results clearly show that the net count rate obtained at 0.5 cm from the granite material was higher than that obtained at a distance of 100 cm. Furthermore, surface activities of granite materials were in the range 35–50 mBq cm^−2^. Additionally, it was found that the ambient dose equivalent rates were not related to surface activity at each measurement point. Granite rocks may contain naturally occurring radionuclides that are strongly enriched in uranium (^238^U) and thorium (^232^Th), compared to soil [21,22] and rocks of basaltic or ultramafic composition [23,24]. Yousef et al. [25] reported that the annual effective dose of granite rock samples was higher than world average values.

### 3.4. In Situ Gamma-Ray Pulse-Height Distribution

**Figure 8:**
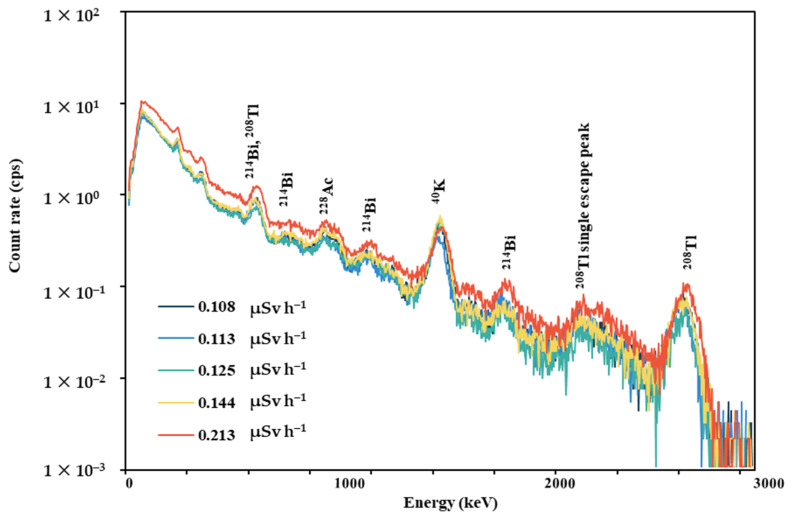
Gamma-ray pulse-height distributions measured at hotspot locations obtained from spot measurements.

After the FDNPP accident in 2011, several artificial radionuclides were measured in many areas using gamma-ray pulse-height distribution [26]. In the current study, the gamma-ray pulse-height distributions for the hotspot locations obtained from spot measurements in Hirosaki City were measured, as shown in Figure 8. There were no photon peaks generated from radioactive cesium (^134^Cs and ^137^Cs), which is a major concern regarding radioactive material contamination in the environment due to the nuclear power plant accident, in these gamma-ray pulse-height distributions. These results demonstrated that there was no substantial radioactive contamination in Hirosaki City (on the Bunkyo campus) due to the FDNPP accident.

### 3.5. Comparison of Dose Rate Distribution Maps between Walking Survey and Spot Measurements

**Figure 9:**
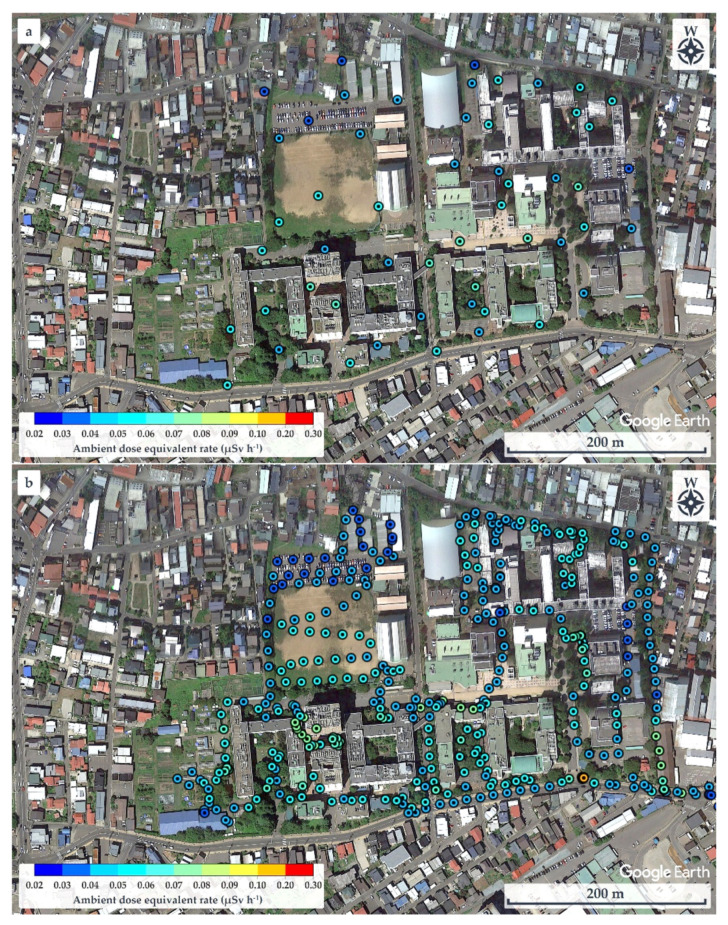
Dose rate distribution maps on Bunkyo campus, Hirosaki University, obtained using (**a**) spot survey technique and (**b**) walking survey.

The walking survey and the spot measurements were performed together on the Bunkyo campus, Hirosaki University, under the established conditions. Figure 9a shows the dose rate distribution map obtained from the spot measurements at 52 points during a total measurement time of about 8 h. The ambient dose equivalent rate was in the range 0.038–0.068 µSv h^−1^ with an average value of 0.052 ± 0.008 µSv h^−1^ and was highest near Building 2 of the Faculty of Science and Technology (GPS: 140.47364° E, 40.58758° N) and lowest in a parking lot (GPS: 140.47193° E, 40.59028° N). In contrast, the dose rate distribution map obtained from the walking survey, shown in Figure 9b with 389 data points, required about 4 h to complete the measurements. The ambient dose equivalent rate was in the range 0.031–0.144 µSv h^−1^, with an average value of 0.052 ± 0.013 µSv h^−1^ and was highest near the University nameplate at the main entrance gate of Hirosaki University (GPS: 140.473865° E, 40.589883° N) and lowest in a parking lot (GPS: 140.472173° E, 40.590268° N).

Comparatively, the spot measurements and the walking survey provided the same average values of the ambient dose equivalent rate. However, the ranges in the dose rates and the locations of the highest dose rates differed. The walking survey covered more detail and depicted points that were not detected during the spot measurement. Notably, the walking survey took only about one-half the time required for the spot measurements.

To evaluate the accuracy of the walking survey technique, the relative errors in measurements were calculated based on Equation (3), using the obtained dose rates from spot measurements as the reference ambient dose equivalent rates:

(3) $$Relative\;error=\frac{D_{walk}-D_{spot}}{D_{spot}}$$

where *D_walk_* and *D_spot_* are the ambient dose equivalent rates obtained from the walking survey and spot measurements, respectively. In this case, *D_walk_* was an average of the walking survey dose rates for the 1–4 data points near a spot measurement point.

**Figure 10:**
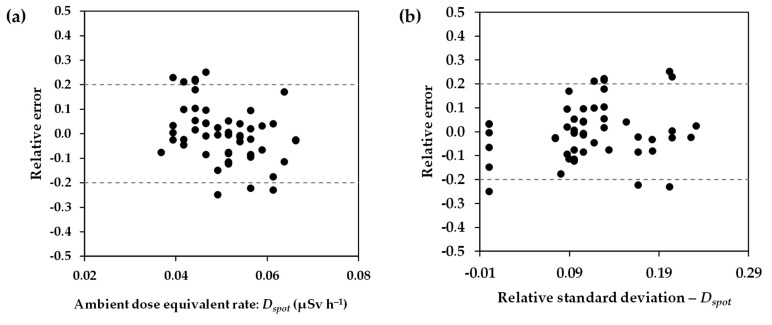
Scatter plots of (**a**) relative error in walking survey measurements against ambient dose equivalent rates of spot measurements and (**b**) relative error in walking survey measurements against relative standard deviation of spot measurements.

The scatter plots of the relative errors in the walking survey measurements (134 points) and the reference ambient dose equivalent rates obtained from spot measurements (52 points) on the Bunkyo campus, Hirosaki University, are shown in Figure 10a. The results showed that most (85% or 44 out of 52) of the relative errors in data points were within ±0.2 with a median value of −0.005. Based on the relative uncertainty in the measurements using the TCS-171 was 15%, the accuracy of the walking survey technique was comparable to that of the spot measurements. A few relative errors (8 of 52 data points) were over ±0.2. Some were caused by low values of ambient dose equivalent rates and statistical fluctuation. Another may be caused by the heterogeneous radiation field around the measurement locations, which showed high relative standard deviation (Figure 10b). The characteristics of these locations were paved surface with buildings and trees/plants. In this study, the gamma rays mainly come from the ground and buildings, whereas those from suspended radon progeny would be a minor component. Shimo et al. [27] reported that 1 Bq m^−3^ of radon progeny concentration (equilibrium equivalent radon concentration) is equivalent to 0.35–0.47 nGy h^−1^. Taking radon concentration (4.4 Bq m^−3^) in Aomori Prefecture [20], equilibrium factor (0.6) in the outdoor environment [17], and the conversion factor (1.25 Sv Gy^−1^) [28] into account, the ambient dose equivalent rate originating from suspended radon progeny was estimated at about 1.1–1.5 nSv h^−1^. The contribution was calculated to be about 2.0–2.7% compared to the average ambient dose equivalent rate observed by the walking survey technique in Hirosaki City. The contribution of suspended radon progeny was negligible to the ambient dose equivalent rates and their distribution in this study.

### 3.6. Comparison of Operation Time between Daytime and Nighttime

**Figure 11:**
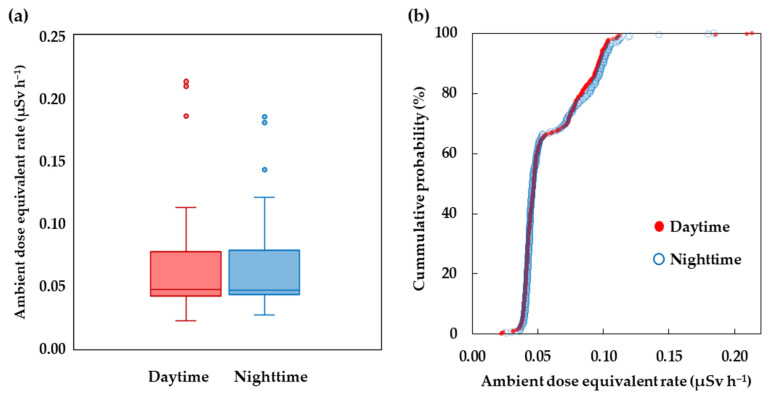
(**a**) Box plot of ambient dose equivalent rates during daytime (red block) and nighttime (blue block). The dotted circles represent outliers of the data. and (**b**) cumulative probability plot of ambient dose equivalent rates in air during daytime and nighttime.

The walking surveys during daytime and nighttime utilized the same walking route around Hirosaki Castle Park. In the daytime, the walking survey was undertaken at 13:00–14:30 p.m. on 15 September 2022. The weather at that time was sunny and clear. The temperature, humidity, and pressure were on average 26.8 ± 1.5 °C, 60.9 ± 3.4%, and 1008.2 ± 1.2 hPa, respectively. The nighttime survey was conducted at 18.30–20.00 p.m. on the same date. The weather at night was characterized by a light wind and a clear sky. The temperature, humidity, and pressure were on average 22.3 ± 1.0 °C, 81.0 ± 5.0%, and 1009.3 ± 1.5 hPa, respectively. The ambient dose equivalent rates obtained during the walking survey during the daytime (395 data points) and the nighttime (319 data points) were compared, as shown in Figure 11a. During the daytime, the ambient dose equivalent rates were in the range 0.022–0.213 µSv h^−1^ with an average value of 0.058 ± 0.022 µSv h^−1^. During the nighttime, the dose rates were in the range 0.026–0.185 µSv h^−1^ with an average value of 0.059 ± 0.023 µSv h^−1^. There was no significant difference between the distribution of dose rates during nighttime and daytime, as shown in Figure 11b, based on the cumulative probability distributions of the ambient dose equivalent rate obtained during the daytime and nighttime surveys. However, the current result does not imply that the operation time for the survey is independent of the measurement time of day. Yoshida et al. [29] reported the variation in the ambient dose rate on fine days rises from night until morning and falls during the day. The variation in radiation dose rate at day and night depends on many factors, such as meteorological conditions, wind speed, and radon concentration. It should be noted that the variation in gamma dose rate during daytime and nighttime is small (a few nanoSievert per hour) compared to the background radiation level, but the variation is not consistent across all locations.

## 4. Conclusions

A walking survey was undertaken using a 3 in × 3 in NaI(Tl) scintillation spectrometer in Hirosaki City, Japan, to estimate and map the distribution of ambient dose equivalent rates in air. The average ambient dose equivalent rates were in the range 0.022–0.213 µSv h^−1^ with an average value of 0.056 ± 0.020 µSv h^−1^ along the walking route. The distribution map obtained from the walking survey was accurate and correlated well with the spot measurements. Furthermore, there was no significant difference between the distribution of dose rates between the daytime and nighttime surveys. Therefore, the air distribution map of ambient dose equivalent rate obtained in this study provided useful information to Hirosaki City residents. The information on radiation exposure levels is essential and interesting for developing a radiological protection culture among citizens and engaging in a dialogue on the issues at stake, such as evacuation orders, decontamination planning, and retrospective risk analyses following a major radionuclide-release incident.

## Figures and Tables

**Table 1:** Hotspot locations with radioactive data of building materials nearby.

Location	Ambient Dose Equivalent Rate (µSv h^−1^)	Net Count Rate (cpm)		Surface Activity (mBq cm^−2^)	Figure
		At 0.5 cm	At 100 cm		
A	0.144	246 ± 55	91 ± 18	50 ± 11	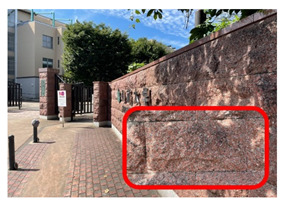
B	0.213	172 ± 12	110 ± 7	35 ± 2	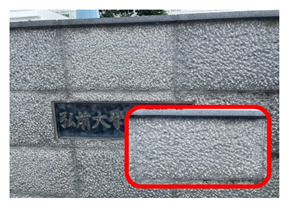
C	0.125	179 ± 26	89 ± 11	36 ± 5	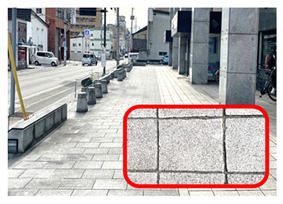
H_2_	0.108	188 ± 62	86 ± 22	38 ± 13	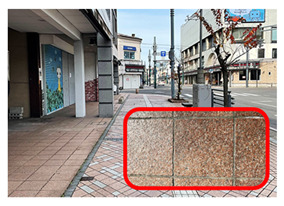
H_3_	0.113	188 ± 11	75 ± 9	38 ± 2	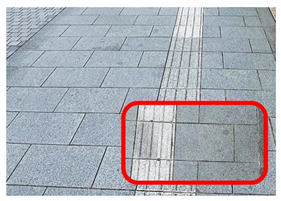

**Figure A1:**
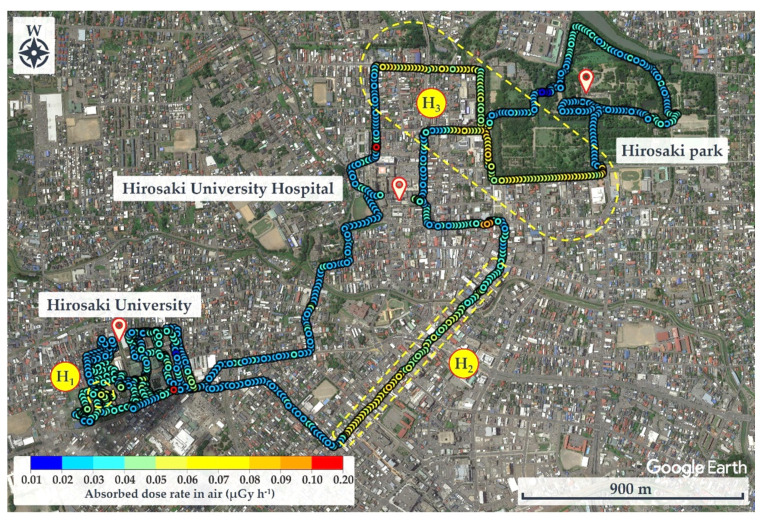
Distribution map of absorbed dose rate in air at Hirosaki City.

## Data Availability

Not applicable.

## Appendix A

Figure A1 shows the distribution map of the absorbed dose rate in air along the walking survey route in Hirosaki City. The estimation of the absorbed dose rate in air used gamma-ray pulse-height distributions that were unfolded based on a 22 × 22 response matrix [10,11].
